# Design, Synthesis and Inhibitory Activity of Photoswitchable RET Kinase Inhibitors

**DOI:** 10.1038/srep09769

**Published:** 2015-05-06

**Authors:** Rubén Ferreira, Jesper R. Nilsson, Carlos Solano, Joakim Andréasson, Morten Grøtli

**Affiliations:** 1 Department of Chemistry and Chemical Engineering, Chalmers University of Technology, SE-412 96 Göteborg, Sweden; 2 Department of Chemistry and Molecular Biology, University of Gothenburg, SE-412 96 Göteborg, Sweden

## Abstract

REarranged during Transfection (RET) is a transmembrane receptor tyrosine kinase required for normal development and maintenance of neurons of the central and peripheral nervous systems. Deregulation of RET and hyperactivity of the RET kinase is intimately connected to several types of human cancers, most notably thyroid cancers, making it an attractive therapeutic target for small-molecule kinase inhibitors. Novel approaches, allowing external control of the activity of RET, would be key additions to the signal transduction toolbox. In this work, photoswitchable RET kinase inhibitors based on azo-functionalized pyrazolopyrimidines were developed, enabling photonic control of RET activity. The most promising compound displays excellent switching properties and stability with good inhibitory effect towards RET in cell-free as well as live-cell assays and a significant difference in inhibitory activity between its two photoisomeric forms. As the first reported photoswitchable small-molecule kinase inhibitor, we consider the herein presented effector to be a significant step forward in the development of tools for kinase signal transduction studies with spatiotemporal control over inhibitor concentration *in situ*.

The use of chemical probes in biology is central to successful translational research. Specific and selective small molecule probes are of key importance for imaging, probing protein mechanisms, and exploring physiology and pathophysiology^1^. They are also at the heart of successful therapeutic intervention in medicine^2^. In contrast to genetic approaches (*e.g*. gene knockouts) that often lack temporal precision (*i.e*. they eliminate the function of the biomolecule at all times and places), small molecules offer dynamic, reversible (non-covalent interactions) and tunable perturbations of biomolecular functions and/or interactions^3^. However, once a small molecule enters the cell, all control over that agent is lost. For example, the intracellular spatial distribution of the reagents as well as the precise timing and endurance of their activity are hard to-manipulate properties. Light can be used as an external stimulus with high temporal and spatial precision. Hence, the use of molecules that change their biological/physicochemical properties upon exposure to light would put the control of the reagent back into the hands of the scientist, even after they have entered the cells.

Here, we have used this approach to control the activity of a REarranged during Transfection (RET) tyrosine kinase receptor by harnessing the isomerization-induced structural changes of a photochromic azobenzene-derived pyrazolopyrimidine unit. Reported examples of photocontrolled kinase inhibition are surprisingly sparse. The so far applied strategies include peptidomimetic approaches^4^,^5^, as well as irreversible activation processes achieved either by using caged inhibitors^6^,^7^ or by introducing photocleavable groups to the kinase itself^8^. The herein presented work instead represents the first example of a photochromic small molecule kinase inhibitor. Photochromic molecules (photoswitches) are a class of chemical compounds that undergo reversible isomerization between two, more or less, thermally stable forms upon light exposure. Fig. 1 exemplifies the structural rearrangements that follow upon isomerization of the azobenzene-derived photoswitch **4**. Given that only one of the two isomeric forms of **4** display activity, the biological process could be controlled in a reversible fashion. Thus, molecular photoswitches are *par excellence* candidates for the abovementioned purposes, and have accordingly been used to photoregulate a multitude of important biologically relevant processes^9^,^10^,^11^,^12^. Azobenzenes form one of the largest and most studied classes of photochromic molecules and are the most widely used photoswitches in biological applications^13^,^14^,^15^,^16^,^17^. The reasons behind this include the ease of synthesis, relatively high photostationary states and isomerization yields, as well as low rate of photodecomposition.

By incorporating azo-bridges into enzyme effectors (*i.e*., inhibitors and activators), it is possible to alter enzyme properties with light, thus altering activity. This strategy has been used to prepare photoswitchable inhibitors of *e.g*. acetylcholinesterase^18^,^19^ and proteases^20^. Previously we reported the design, synthesis and biological evaluation of a small library of 3-substituted pyrazolopyrimidines that inhibit the RET kinase in low nanomolar concentrations (down to 8 nM) *in vitro*^21^. Compound **1** displayed efficient inhibition *in vitro* and good kinase selectivity. Moreover, it was shown to inhibit GDNF-induced RET phosphorylation of ERK172 in MCF-7 breast cancer cells at concentrations as low as 100 nM^21^. With **1** as the inspiration, we designed a series of photoswitchable pyrazolopyrimidine chromophores to potentially gain photonic control over the activity of RET. We hypothesized that the RET kinase domain would not tolerate the inhibitor in the *Z*-form (Fig. 1b), leading to a design strategy focused on incorporation of the photoswitchable unit in the 3-position of the pyrazolopyrimidine scaffold.

Also, knowing the RET kinase preference for **1** over several analogs with substituents on the phenyl ring, we opted for an unsubstituted switching unit^21^. The already synthesized **2** composed a natural starting point, carrying a known photoswitching unit (the stilbene unit) and having documented inhibitory ability toward the RET kinase^21^. Further synthetic efforts also yielded **3** and **4** (Fig. 2) from the azobenzene family.

## Results and Discussion

### Synthesis and photophysical characterization

**Figure 3:**
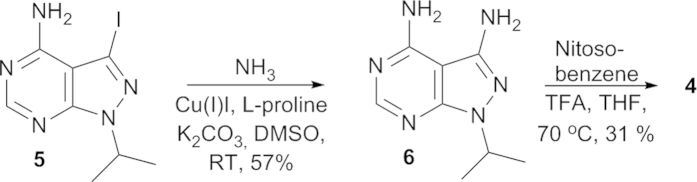
Synthesis of photoswitchable kinase inhibitor **4**.

Compound **2** was synthesized using Suzuki-Miyaura cross coupling reaction as previously reported^21^. The classical methods for the synthesis of azo compounds are the azo coupling reaction, the Mills reaction and the Wallach reaction^22^. The mechanism of the Mills reaction involves the attack of aniline on the nitroso derivative in acid media that leads to azobenzene after dehydration of the intermediate^23^. Here we applied the Mills reaction to the synthesis of azo derivative **4**. The designed compound was readily amenable to two-step synthetic route (Fig. 3). The 3-iodo-1-isopropyl-1H-pyrazolo[3,4-d]dypirimidine-4-amine **5** was used as starting material and obtained as described previously^21^. Intermediate **6** was achieved with copper catalyzed amination at room temperature upon addition of base using L-proline as ligand and aqueous ammonia as nitrogen source^24^. The final step of the synthesis is the introduction of the azo functionality using the Mills reaction. The acid catalyzed reaction using nitrosobenzene targeted compound **4** in a moderate yield. Double or concurrent azo formation to competing nucleophilic attack of the second amino functionality in the starting material was not observed, likely due to lower reactivity of the pyrimidine amino group.

As for light-induced modulation, the molecular design must be such that a significant difference in target affinity is attained between the two photoisomeric forms. In addition, and of equal importance, is a high degree of photo-conversion (to significantly change the ratio active *vs*. passive form) and stability towards thermal isomerization (to maintain the ratio over time). Prolonged and repeated operation in a biological matrix also requires the photoswitch to be stable towards photo- as well as solvent-induced degradation (*e.g*. hydrolysis). UV/Vis spectroscopy is well suited to assess several of these criteria. In water, compounds **2**, **3**, and **4** exhibit absorption maxima at 313 nm, 375 nm and 349 nm, respectively (see Figs. S7, S8, and 4 for spectra).

**Figure 4:**
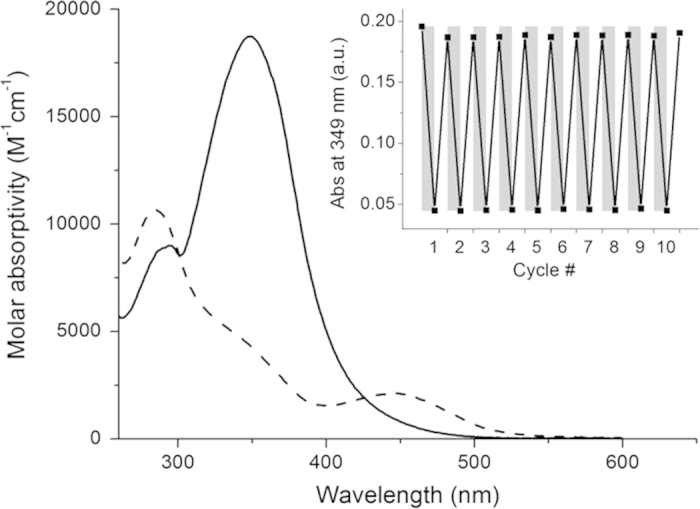
UV/Vis absorption spectra and photoswitching of **4**. The as-dissolved *E*-**4** (solid line) was subjected to 3 min 365 nm light, yielding a photostationary distribution (PSD) of 87/13 [*Z*-**4**]/[*E*-**4**] (dashed line). Inset: Photoswitching of **4** (10 μM) monitored at λ = 349 nm, starting with pure *E*-**4** followed by alternating irradiation periods of 3 min 365 nm (gray bars) and 3 min 503 nm (white bars).

As for photoinduced reactions, the compounds differ significantly. Attempted photoswitching of stilbene-derived **2** using 302 nm resulted in decreased absorption for all wavelengths. After irradiation, left in the dark, a partial recovery of the spectrum is noted (see Fig. S7 for spectra). This recovery, however, did not follow the spectral trajectory observed in the photoinduced process. We interpret this behavior as an irreversible photoreaction taking place prior to and/or following photoinduced *E*-**2** → *Z*-**2** isomerization. The azobenzene-substituted compounds demonstrated more favorable switching characteristics. Compared to **2**, the absorption spectrum of **3** and **4** are red-shifted, allowing for lower energy photons to be used for switching. Exposing **3** to 365 nm light caused a decrease in the absorption band centered at 375 nm in concurrence with an increase in absorption of *Z*-**3** for *λ* < 300 nm. In the dark, the sample promptly reverted (*τ* = 2.0 min at 37 °C, Fig. S9) to the as-dissolved *E*-**3** form. The fast thermal isomerization *Z*-**3** → *E*-**3** prevented determination of a photostationary distribution (PSD) for **3**. Similarly to **3**, compound **4** experienced a decrease in the main absorption band centered at 349 nm upon 365 nm light exposure with a time constant *τ* = 21 s (700 μW/cm^2^, see Figs. S12 and S13 for details on photoswitching). For **4**, however, the spectral changes were more pronounced, and accompanied by the emergence of a new band centered at 443 nm which we attribute to *Z*-**4**. Collectively, these spectral features are known as the hallmark signs of azobenzene photoswitching. In contrast to **3**, the ensuing dark process was significantly slower (*τ* = 9.7 h at 37 °C *vs*. 2.0 min for **3**, Fig. S15), indicating a decidedly more thermally stable *Z*-isomer. The photostationary distribution reached using 365 nm light was determined with HPLC to 87% *Z*-form. The reverse reaction (*Z*-**4** → *E*-**4**) can also be triggered photonically using visible light (here: 503 nm, 14 mW/cm^2^, *τ* = 37 s, Fig. S13). This *E* ⇌ *Z* switching cycle could be repeated 10 times without any signs of photo-fatigue (Fig. 4, Inset). Both the photoinduced and thermal processes proceeded with clear isosbestic points at 299 nm and 426 nm, indicating clean conversion between the two isomeric forms. Hydrolytic stability was assessed for all compounds by placing the as-dissolved samples in the dark at 37 °C. No changes in absorption were detected over five days under these conditions (data not shown), indicating excellent resistance to hydrolysis.

### Cell-free incubation

**Figure 5:**
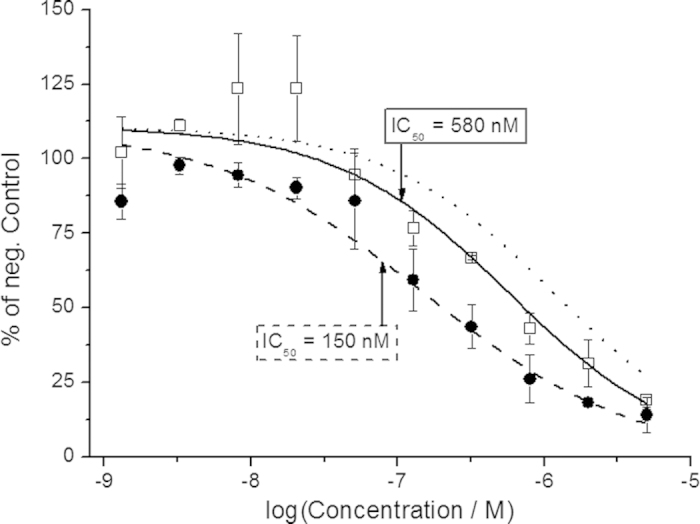
Cell-free RET incubation with **4**. RET-induced ATP turnover was monitored *via* luminescence intensity. The activity readout of *E*-**4** (solid circles) and after light-induced *E*-**4** → *Z*-**4** conversion (3 min 365 nm, hollow squares) was referenced to a negative control (without inhibitor). Fitting to the Hill-equation renders IC_50_-values of 150 nM and 580 nM for *E*-**4** (dashed line) and photo-enriched *Z*-**4** (solid line), respectively. Error bars are mean ± standard deviation of duplicate samples. The dotted line illustrates the minimum inhibitory effect of **4**, given the 87% *Z*-form PSD, and assuming no inhibitory effect of *Z*-**4**. See Methods section for the applied fitting procedure.

The RET tyrosine kinase domain has been isolated and adapted to a substrate initiated ATP-to-ADP conversion assay with activity-dependent luminescence readout, facilitating *in vitro* screening of RET kinase activity and inhibition thereof^25^,^26^. Having displayed superior characteristics in terms of photoswitching and thermal stability (*vide supra*), **4** was selected for biological evaluation. Compound **4** was included in two parallel preparations with RET kinase, substrate (IGFlRtide), and co-factors (see Methods section below for incubation details). One preparation was at this point exposed to light (365 nm, 3 min), thus converting as-dissolved *E*-**4** → *Z*-**4**, while the other was kept in the dark. Following addition of ATP and incubation at room temperature for 30 min, the relative ATP turnover was assessed for in total 10 concentrations of **4**. The resulting dose-response curves (Fig. 5) reveal a significant difference in inhibitory capability between the two photo-isomers, with IC_50_-values of 150 nM and 580 nM for *E*-**4** and photo-enriched *Z*-**4**, respectively (fitting details are outlined in the Methods section). To rule out any distortive effects of the light itself on the enzyme assay, a set of control experiments (without inhibitor) with up to 15 min 365 nm exposure were conducted, showing no light-induced changes in enzymatic ATP turnover (Fig. S16).

### Live-cell incubation

**Figure 6:**
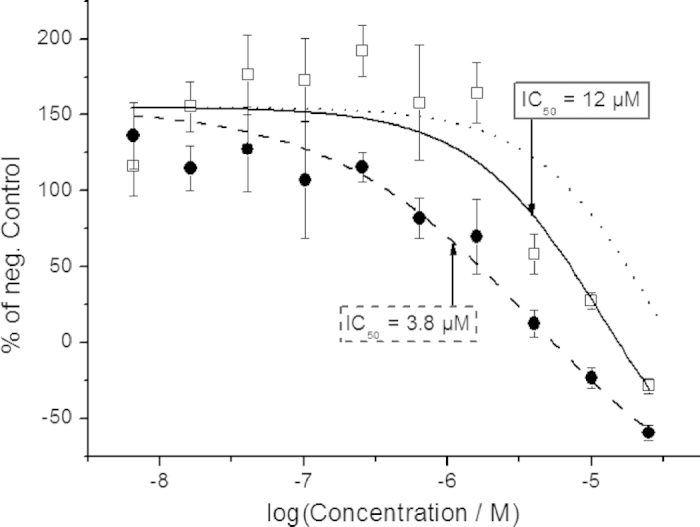
Live-cell RET incubation with **4**. RET-activity was monitored *via* luminescence intensity. The activity readout of *E*-**4** (solid circles) and after light induced *E*-**4** → *Z*-**4** conversion (3 min 365 nm, hollow squares) was referenced to a negative control (without inhibitor). Fitting to the Hill-equation renders IC_50_-values of 3.8 μM and 12 μM for *E*-**4** (dashed line) and photo-enriched *Z*-**4** (solid line), respectively. Error bars are mean ± standard deviation of quadruplicate samples. The dotted line illustrates the minimum inhibitory effect of **4**, given the 87% *Z*-form PSD, and assuming no inhibitory effect of *Z*-**4**. See Methods section for the applied fitting procedure.

Encouraged by the clear difference in inhibitory effect observed in the isolated enzyme, we turned to demonstrate the effect also in a living system. Thus, the inhibitory activity of *E*-**4** and *Z*-**4** was evaluated in a cell-based functional assay, which utilizes β-galactosidase-based enzyme fragment complementation technology to produce enzyme-activity correlated luminescence readout^27^,^28^,^29^. Compound **4** was added to live cells expressing the RET kinase (see Methods section for incubation details) in two identical preparations, followed by preincubation for 3 h at 37 °C. Subsequently, one preparation was exposed to light (365 nm, 3 min), converting *E*-**4** → *Z*-**4**, while the other was kept in the dark. Addition of the GDNF-family growth factor Neurturin and incubation for 3 h at 22 °C was followed by induction of luminescence signal using the supplemented detection reagents. The incubations were performed with 10 concentrations of **4**. The dose-response curves shown in Fig. 6 provides IC_50_-values of 3.8 μM and 12 μM for *E*-**4** and photo-enriched *Z*-**4**, respectively. In Fig. 6, it can be seen that the enzyme activity can be suppressed beyond what is observed when no growth factor was added, leading to negative values. This is likely due to constitutive (growth factor independent) enzyme activity in the cells, which has previously been reported for similar cell lines^28^. The light tolerance of the live-cell assay without inhibitor was assessed, showing no apparent loss in RET-activity for up to 15 min 365 nm exposure (Fig. S16). To evaluate the change in efficacy caused by exchanging the parent ethyne bridge with the azo bridge (compound **1 → 4**), we performed the cell-free and live-cell incubations also with **1** (Figs. S10, S11). It is concluded that the structural modification increases the IC_50_-values by a factor 2.1 and 8.1 for the cell-free and live-cell assay, respectively. A general increase is expected, while the observed difference between the two assays may be attributed to differences in cell uptake between **1** and **4**. Reductive degradation has been reported as a potentially limitating factor for *in vivo* use of azobenzene photoswitches^30^. To elucidate the impact of this in the live-cell assay, the photochromic performance of **4** in the presence of glutathione was examined. No significant degradation of **4** was seen under the applied conditions (Fig. S14).

The photostationary distribution of **4** (87% *Z*-form in water), limits the maximum attainable difference in inhibitory effect to a factor of 7.7, assuming no inhibitory effect of *Z*-**4** (see dotted lines in Figs. 5 and 6). It has been previously suggested, however, that photostationary distributions different from those observed in water may be obtained in a cellular environment, as a result of changes in bulk polarity and/or steric congestion in the enzyme^31^,^32^. The experimentally observed difference in inhibition of **4** with *vs*. without light is 3.8-fold and 3.2-fold in the enzyme- and live-cell experiments, respectively. The discrepancy (observed: 3.8 and 3.2 *vs*. PSD-max: 7.7) may alternatively be explained by thermal *Z* → *E* isomerization during incubation. Based on our initial characterization of **4** in water, the observed thermal rate should not significantly change the isomeric distribution (87% *Z*-form → *ca*. 80% for 3 h incubation at 22 °C); Also, we did not observe any major variations in the rate of the thermal process in the less polar solvents DMSO and toluene (data not shown). It has been suggested that receptor environments can influence the rate of thermal *Z* → *E* isomerization of azobenzenes^33^. This, however, is unlikely to explain the observed discrepancy, as the thermal isomerization rate in the enzyme is more likely to be reduced. Last, but not least, it is acknowledged that the *Z*-isomer may contribute to the inhibition, although it would require a significant degree of active site plasticity to accommodate this isomer.

## Conclusions

We have presented the design, synthesis, and photophysical/biological characterization of azobenzene-derived photoswitches aiming at photocontrolled RET kinase inhibition. This study represents the first reported example of a photoswitchable small molecule kinase inhibitor. Results from cell-free as well as live-cell experiments clearly show that photoisomerization from the *E*-isomer to the *Z*-isomer was readily achieved *in situ* with a concomitant decrease in the inhibitory effect. Studies aimed at photocontrolled regulation of biological activity are often motivated by potential clinical applications. We anticipate, however, that the results presented in this study will find more immediate value in the development of research tools for resolving quantitative and dynamic aspects of kinase signal transduction. In addition, other reported kinase inhibitors containing functional groups that can be regarded as isosteres of an azo-bridge, could probably be converted to photoswitchable kinase inhibitors using the same approach as herein described.

## Methods

### Spectroscopic methods and instrumentation

Steady state absorption measurements were carried out on a Cary Bio 50 UV/Vis spectrometer equipped with a Varian PCB 1500 Water Peltier System thermostat for temperature control. Solvent was mQ-water or 1:99 DMSO:water mixture, unless otherwise stated. UV-induced isomerizations were performed using a hand-held UVP UV-lamp model UVGL-25 delivering a power density of 700 μW/cm^2^ (for 365 nm) or a hand-held UVP UV-lamp model UVM-57 delivering a power density of 1.5 mW/cm^2^ (for 302 nm). Green light (503 nm) was achieved using a 500 W Xe lamp equipped with a hot mirror (*A* = 1.9 at 900 nm) to reduce IR intensity and an interference filter (*T*_*max*_ at 503 nm, *FWHM*: *ca*. 20 nm), resulting in a power density of 14 mW/cm^2^. Sample volume was *ca*. 2.5 mL. Reversed phase HPLC separation with UV-detection at *λ* = 300 nm (isosbestic point) was used for determining the photo stationary distribution of **4**.

### Cell-free incubations

Human recombinant RET expressed in Sf9 insect cells (Specific Activity: 220 nmol^-1^min^-1^mg^-1^), substrate (IGFlRtide), and ATP were purchased in an assay ready kit^34^ from Promega (Promega Corporation, Madison WI 53711 USA) and used as received. Incubations were performed in 40 mM Tris buffer (pH 7.4, 1vol% DMSO) supplemented with 50 μM DTT and 20 mM MgCl in a white flat-bottom 96-well plate. Incubation volume was 25 μL. Brief procedure; Kinase (0.8 μg/mL), substrate (40 μg/mL), ATP (50 μM) and inhibitor were combined and incubated for 30 min at room temperature in the dark, with shaking. An [ADP]-correlating luminescence signal was induced using the provided luciferase-based detection reagents. Luminescence was recorded on a BMG Labtech Fluostar Omega luminometer. All incubations were done in duplicates.

### Live-cell incubations

PathHunter express c-RET-GFRα2 functional assay^28^ and recombinant human Neurturin were purchased from DiscoveRx (DiscoveRx Corporation, Fremont CA 94538 USA) and used according to instructions. Incubations were performed in the provided assay buffer (1vol% DMSO) in a white flat/clear-bottom 96-well plate. Incubation volume was 110 μL. Brief procedure; Cryopreserved cells were thawed and diluted to *ca*. 100 000 cells/mL, followed by plating and acclimatization in 37 °C, 5% CO_2_, humidified, environment for 48 h. Thereafter, the inhibitor was added and the plate was returned to 37 °C for 3 h. The cells were then stimulated with the GDNF-family growth factor Neurturin (at EC_80_, determined to 15 ng/mL) and incubated 3 h at 22 °C in the dark. An activity-correlating luminescence signal was induced using the detection reagent provided in the kit according to the recommended protocol. Luminescence was recorded on a BMG Labtech Fluostar Omega luminometer. All incubations were performed in quadruplicates.

### Fitting of dose-response data

Positive and negative control experiments with and without light exposure (3 min, 365 nm) were run in parallel to the inhibitor incubations. The positive control was identical to the inhibitor experiment but with buffer additions instead of ATP, and Neurturin for the cell-free and live-cell incubation, respectively. The negative control was identical to the inhibitor experiment but with inhibitor diluent (DMSO) addition instead of inhibitor. The intensity from the positive control was subtracted from the corresponding inhibitor incubation, followed by normalization to the negative control reading. The resulting luminescence intensities (*I*) at the applied inhibitor concentrations (*C*) were fitted to equation (1):
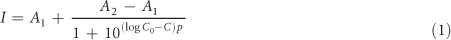


A global, error-weighted (instrumental) fit was performed by making the top- (*A*_*2*_) and bottom (*A*_*1*_) asymptotes shared parameters for the irradiated and non-irradiated data sets. The authors opted for shared asymptotic parameters, as the inhibitor screenings were performed with the same preparation and the luminescence signals were referenced to the same negative and positive controls. The fitted parameters and associated errors from the enzyme- and live-cell assays with photoswitch **4** can be found in the Supporting Information (Table T1 and Table T2, respectively).

## Author Contributions

C.S. and R.F. synthesized the compounds. R.F. also contributed to the interpretation of the biological assay data. J.R.N. carried out the spectroscopic characterization, the biological testing and interpreted the data. M.G. and J.A. developed the experimental strategy. J.R.N. and M.G. wrote the paper. J.R.N., J.A., M.G. and R.F. reviewed the paper.

## Additional Information

**How to cite this article**: Ferreira, R. *et al.* Design, Synthesis and Inhibitory Activity of Photoswitchable RET Kinase Inhibitors. *Sci. Rep*. **5**, 09769; doi: 10.1038/srep09769 (2015).

## Supplementary Material

Supplementary Information

## Figures and Tables

**Figure 1:**
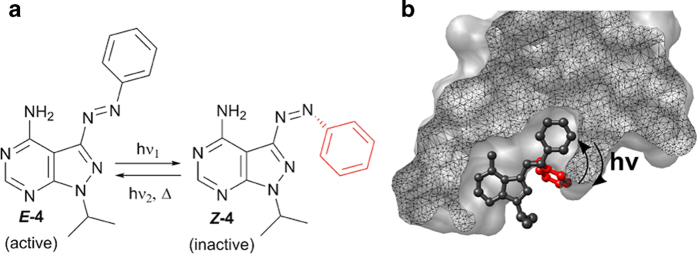
(**a**) Structure and isomerization of the azobenzene-derived photoswitch **4**. (**b**) Surface representation of the RET kinase domain with the photoswitchable kinase inhibitor **4** in the *E*-form (black) and *Z*-form (red). It is clear that the geometry of the *Z*-form hinders binding to the kinase domain.

**Figure 2:**
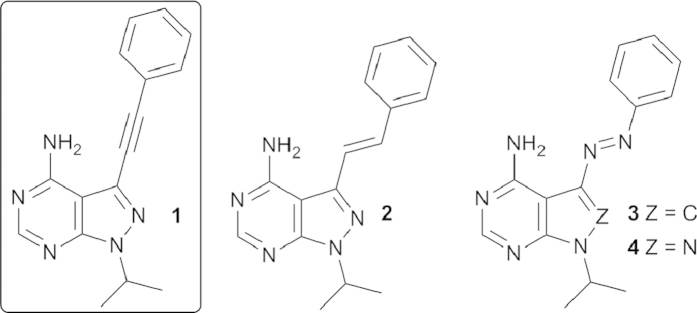
Pyrazolo-pyrimidine RET kinase inhibitor **1** and potential photo-switchable derivatives (**2**, **3** and **4**).
