# Supplementary material for: Design, Synthesis and Inhibitory Activity of Photoswitchable RET Kinase Inhibitors
Source: Sci Rep. 2015 May 6;5:9769. doi: 10.1038/srep09769 (PMC4421829; doi:10.1038/srep09769)
Supplement: Supplementary Information [file srep09769-s1.pdf]

Supporting Information

Design, Synthesis and Inhibitory Activity of Photoswitchable RET Kinase Inhibitors

Rubén Ferreira,[a] Jesper R. Nilsson,[a] Carlos Solano,[b] Joakim Andréasson,[*a] and Morten Grøtli[*b]

[a]Department of Chemistry and Biological Engineering, Chalmers University of Technology, SE-412 96 Göteborg, Sweden, E-mail: a-son@chalmers.se
[b]Department of Chemistry and Molecular Biology, University of Gothenburg, SE-412 96 Göteborg, Sweden, E-mail: grotli@chem.gu.se

Content

Entry ... Page

1. Synthesis of photoswitchable inhibitors and precursors ... S1-S6
    1.1 Synthesis of **3** ... S1-S2
    1.2 Synthesis of **4** ... S3-S4
    1.3 Synthesis of **6** ... S5-S6
2. Fitting parameters for dose-response data ... S7
3. Supplementary figures ... S8-S13
    3.1 UV/Vis spectra of **2** ... S8
    3.2 UV/Vis spectra and thermal isomerization kinetics of **3** ... S9
    3.3 Dose-response data for **1** ... S10
    3.4 Photo- and thermal kinetics of **4** ... S11-S12
    3.5 UV-tolerance of RET incubation assays ... S13
4. References ... S14

1. Synthesis of photoswitchable inhibitors and precursors

1.1 Synthesis of 3

7-isopropyl-5-(phenyldiazenyl)-7H-pyrrolo[2,3-d]pyrimidin-4-amine (3): In an oven-dried 20 ml microwave vessel 5-(phenyldiazenyl)-7H-pyrrolo[2,3-d]pyrimidin-4-amine[1] (0.20 g, 0.84 mmol) and cesium carbonate (0.55 g, 1.68 mmol) were suspended in dry N,N-dimethylformamide (10 mL). 2-Iodopropane was added (0.092 mL, 0.92 mmol) and the vessel was sealed and submitted to the microwave. With a pre-stirring of 30 seconds the mixture was heated to 90 °C for 20 minutes. The solvent was evaporated by co-distillation with toluene and the crude mixture was taken up with $CHCl_3$ and purified by silica gel column chromatography using a gradient of $CHCl_3$ with 0-10% MeOH, evaporated and dried under vacuum. The target compound was obtained as a yellow powder (0.139 g, 59%). ^1H NMR ($CDCl_3$, 400 MHz) δ (ppm) = 8.62 (s, br, 1H), 8.32 (s, 1H), 7.92 (s, 1H), 7.76-7.78 (m, 2H), 7.46-7.51 (m, 2H), 7.39-7.43 (m, 1H), 5.66 (s, br, 1H), 5.10 (sept, 1H, J = 6.8 Hz), 1.58 (d, J = 6.8 Hz, 6H). ^{13}C NMR ($CDCl_3$) δ (ppm) = 158.0, 153.9, 152.4, 151.7, 136.0, 130.0, 129.4, 129.3, 121.8, 95.0, 46.5, 22.8. MS-ESI (m/z): $[M+H]^+$ calcd for $C_{15}H_{16}N_6$, 281.1; found 281.1. Anal. Calcd for $C_{15}H_{16}N_6$: C, 64.27; H, 5.75; N, 29.98. Found: C, 64.30; H, 5.76; N, 29.99.

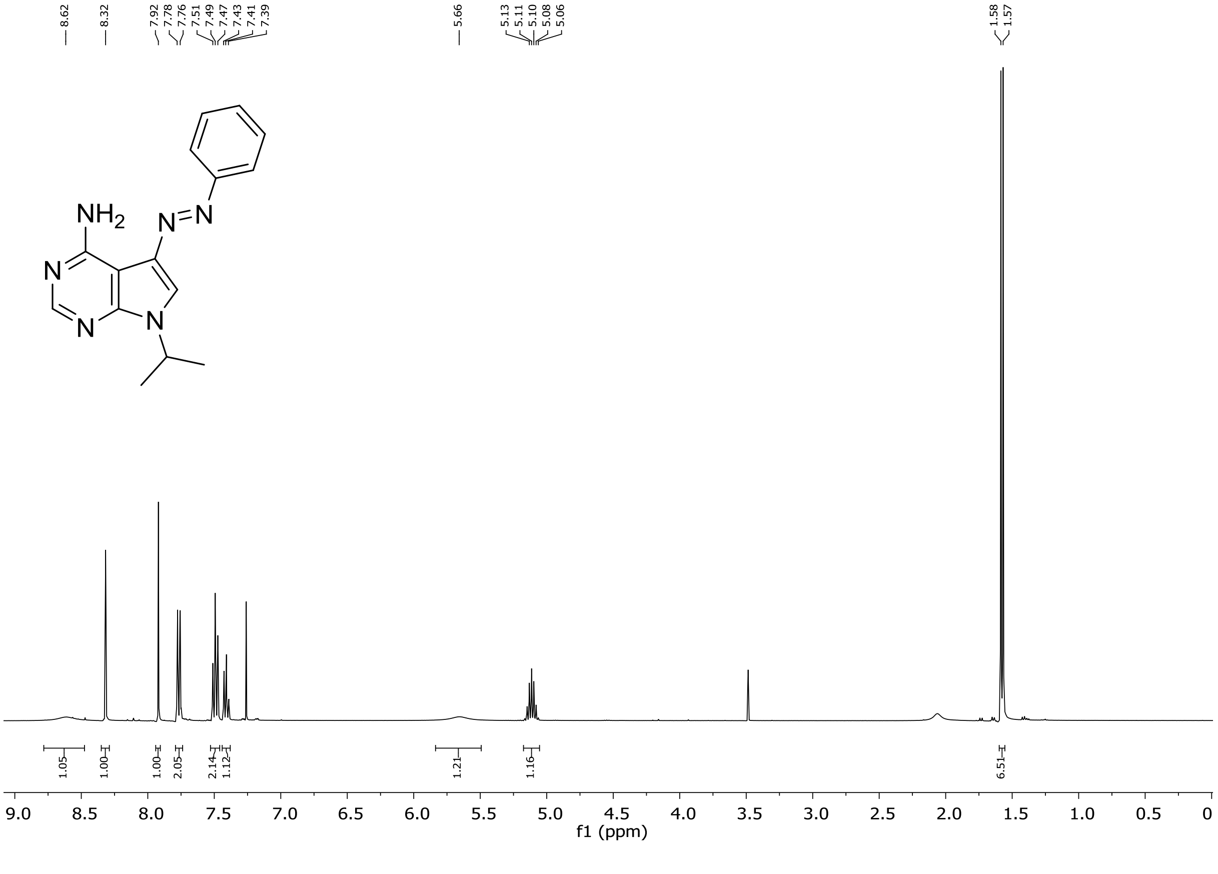

Figure S1. ^1H NMR spectrum of **3**.

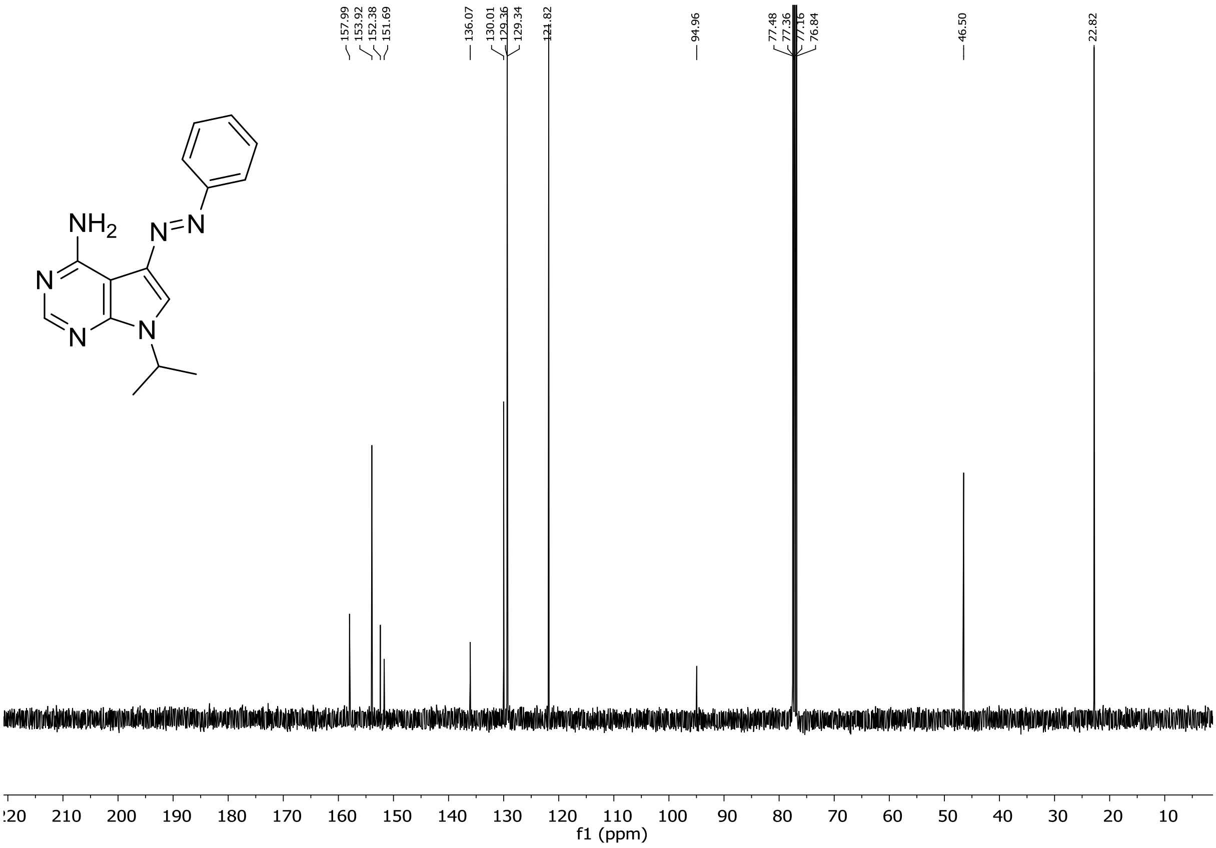

Figure S2. ^{13}C NMR spectrum of **3**.

1.2 Synthesis of 4

1-isopropyl-3-(phenyldiazenyl)-1H-pyrazolo[3,4-d]pyrimidin-4-amine (4): 3-amino-1-isopropyl-1H-pyrazolo[3,4-d]dypirimidine-4-amine **6** (0.13 g, 0.67 mmol) and nitrosobenzene (0.025 g, 0.23 mmol) were dissolved in dry THF (5 mL) under nitrogen atmosphere. Trifluoroacetic acid (0.047 mL, 0.62 mmol) was added and the mixture was stirred for 48 h at 70˚C. The reaction mixture was quenched by adding triethylamine. The solvent was evaporated under reduced pressure and the resulting crude product purified by preparative HPLC (Eluent A: H_2O + 0.1% TFA; Eluent B: ACN + 0.1% TFA) with a 20-80% ACN gradient, run over 20 minutes. The solvent was evaporated, affording compound **4** as yellow powder (0.059 g, 31%). ^{1}H NMR ($CDCl_3$, 400 MHz) δ (ppm) = 9.38 (s, br, 1H), 8.24 (s, 1H), 7.98-8.00 (m, 2H), 7.56-7.62 (m, 3H), 5.29 (sept, J = 6.8 Hz, 1H), 1.69 (d, J = 6.8 Hz, 6H). ^{13}C NMR ($CDCl_3$) δ (ppm) = 157.3, 153.9, 151.7, 151.3, 147.2, 133.8, 129.9, 123.4, 92.1, 51.2, 22.0. MS-ESI (m/z): $[M+H]^+$ calcd for $C_{14}H_{15}N_7$, 282.1; found 282.3. HRMS *m/z* $[M + H]^+$ calcd for $C_{14}H_{15}N_7$: 282.1467. Found: 282.1469. Anal. Calcd for $C_{14}H_{15}N_7$: C, 59.77; H, 5.37; N, 34.85. Found: C, 59.79; H, 5.36; N, 34.36.

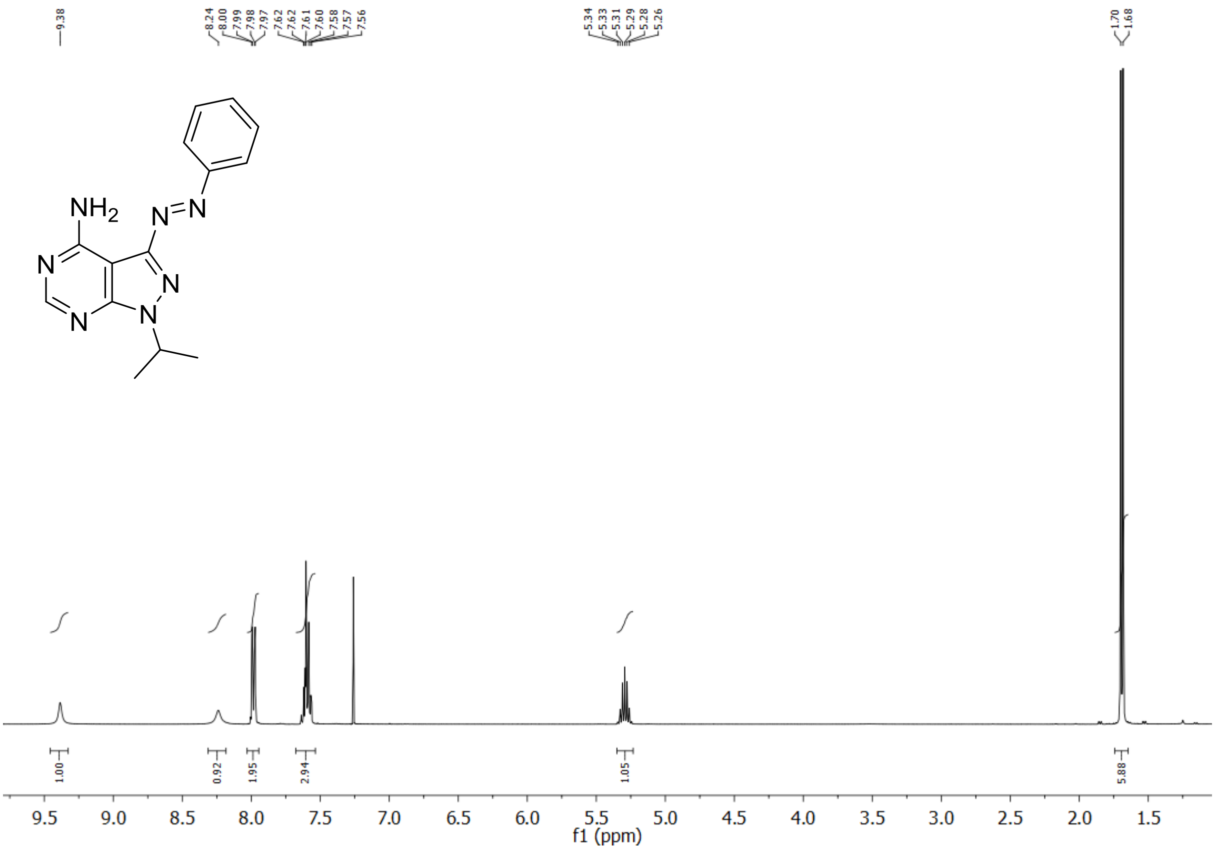

Figure S3.^{1}H NMR spectrum of **4**.

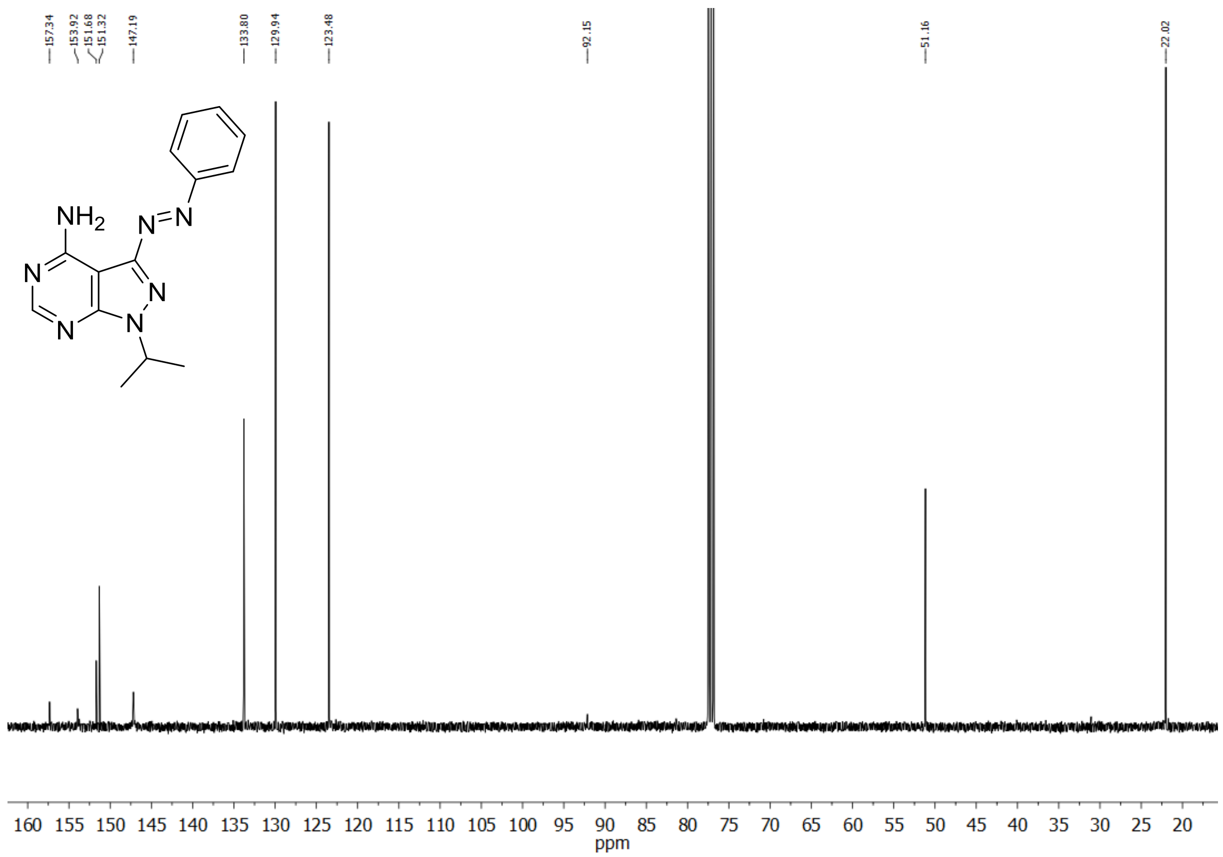

Figure S4. ^{13}C NMR spectrum of **4**.

1.3 Synthesis of 6

Synthesis of 3-amino-1-isopropyl-1H-pyrazolo[3,4-d]dypirimidine-4-amine (6): 3-iodo-1-isopropyl-1H-pyrazolo[3,4-d]dypirimidine-4-amine **5**[2] (0.35 g, 1.14 mmol) copper(I)iodide (0.04 g, 0.23 mmol) L-proline (0.05 g, 0.46 mmol) and potassium carbonate (0.24 g, 1.71 mmol) were suspended in DMSO (4.0 mL). Ammonia (aq.) solution (28%, excess) was added to the solution, and the mixture was stirred under nitrogen for 15 h at room temperature. The reaction mixture was diluted in water and extracted three times with EtOAc. The combined organic phases were dried with anhydrous sodium sulfate, filtered, concentrated under reduced pressure. The resulting product was purified by washing with fresh EtOAc affording compound **6** as white solid (0.13 g, 57%). ^{1}H NMR (DMSO-d6, 400 MHz) δ (ppm) = 7.96 (s, 1H), 7.14 (s, br, 2H), 5.65 (s, br, 2H), 4.76 (sept, J = 6.7 Hz, 1H), 1.31 (d, J = 6.8 Hz, 6H). ^{13}C NMR (DMSO-d6) δ (ppm) = 157.8, 155.6, 152.4, 147.0, 89.8, 46.3, 21.6. MS-ESI (m/z): $[M+H]^+$ calcd for $C_8H_{12}N_6$, 193.1; found 193.1. Anal. Calcd for $C_8H_{12}N_6$: C, 49.99; H, 6.29; N, 43.72. Found: C, 50.01; H, 6.30; N, 43.74.

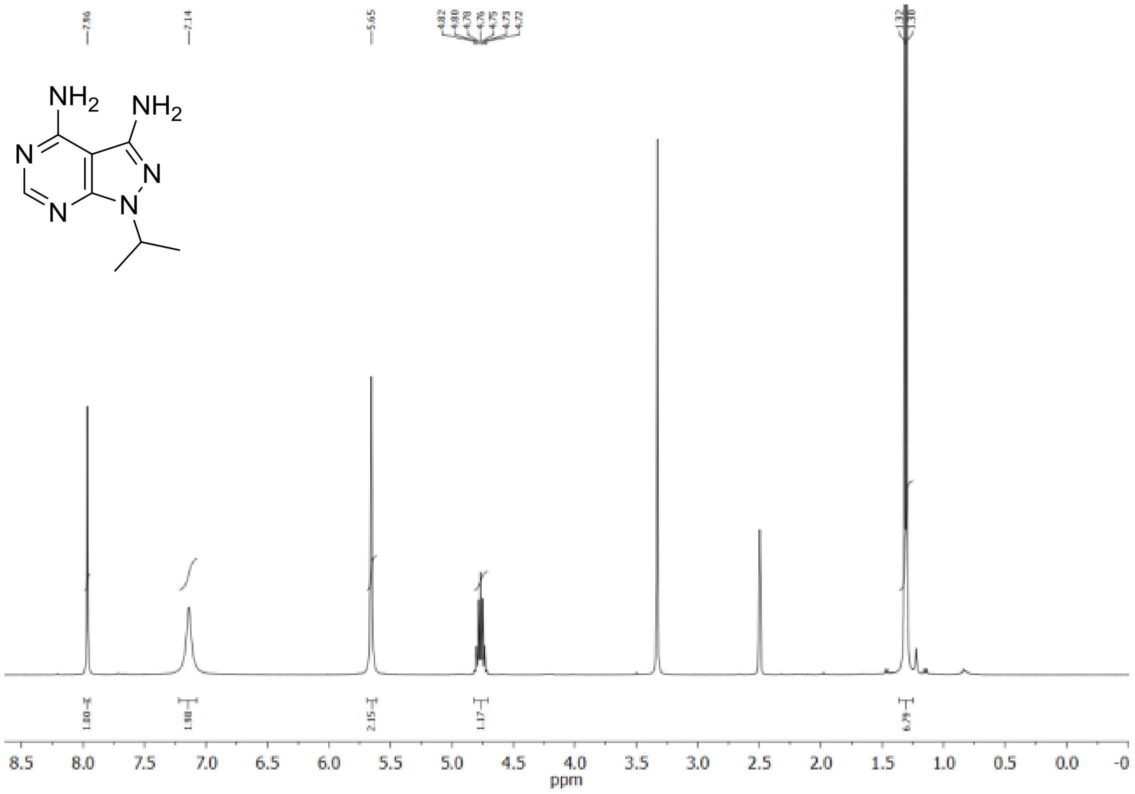

Figure S5.^{1}H NMR spectrum of **6**.

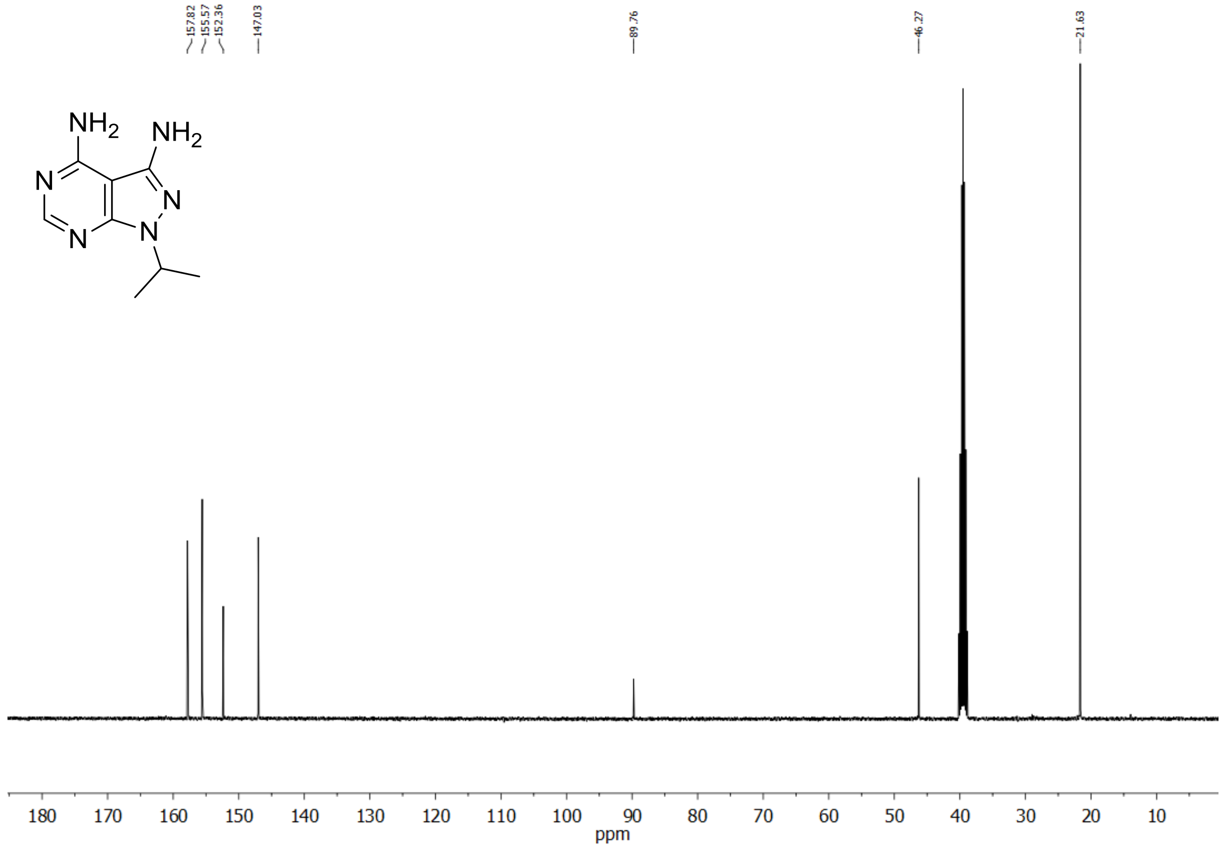

Figure S6. ^{13}C NMR spectrum of **6**.

2. Fitting parameters for dose-response data

Luminescence intensities (I) at the applied inhibitor concentrations (C) were fitted to equation (1):

$$I = A_1 + \frac{A_2 - A_1}{1 + 10^{(\log C_0 - C)p}} \tag{1}$$

Table T1. Fitted parameters and associated errors from the cell-free RET incubation (Fig. 5 in the manuscript).

	E-**4**	SEǂ (*E*-**4**)	Photo-enriched *Z*-**4**	SEǂ (*Z*-**4**)
A_1*	-0.9102	13.68	-0.9102	13.68
A_2*	110.7	3.966	110.7	3.966
$\log(C_0)$	-6.820	0.2493	-6.241	0.1543
p	-0.6040	0.1422	-0.7376	0.2018

*Shared parameter.
ǂ SE = Standard error. Adjusted R^2 for the global fit was 0.9910.

Table T2. Fitted parameters and associated errors from the live-cell RET incubation (Fig. 6 in the manuscript).

	E-**4**	SEǂ (*E*-**4**)	Photo-enriched *Z*-**4**	SEǂ (*Z*-**4**)
A_1*	-124.9	56.44	-124.9	56.44
A_2*	154.9	12.40	154.9	12.40
$\log(C_0)$	-5.417	0.3179	-4.912	0.1851
p	-0.6162	0.1866	-0.9456	0.2761

*Shared parameter.
ǂ SE = Standard error. Adjusted R^2 for the global fit was 0.9614.

3. Supplementary figures

3.1 UV/Vis spectra of 2

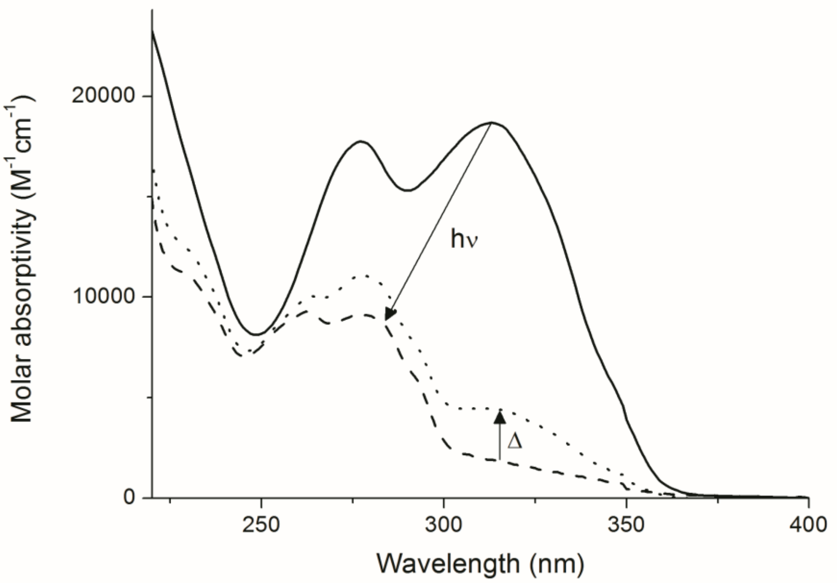

Figure S7. UV/Vis absorption spectra of **2** (7 µM) in mQ-water. As-dissolved *E*-**2** (solid line) was subjected to 9 min 302 nm light (PSS, dashed line). Left in the dark (18 h, 22 °C), the spectrum makes a partial recovery (dotted line).

3.2 UV/Vis spectra and thermal isomerization kinetics of 3

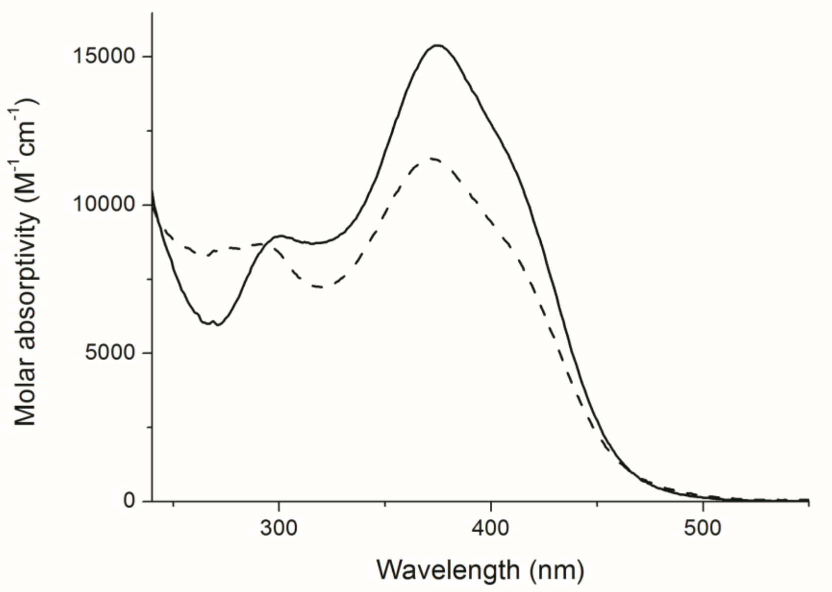

Figure S8. UV/Vis absorption spectra of **3** (30 µM) in mQ water. As-dissolved *E*-**3** (solid line) was subjected to 3 min 365 nm light (PSS, dashed line).

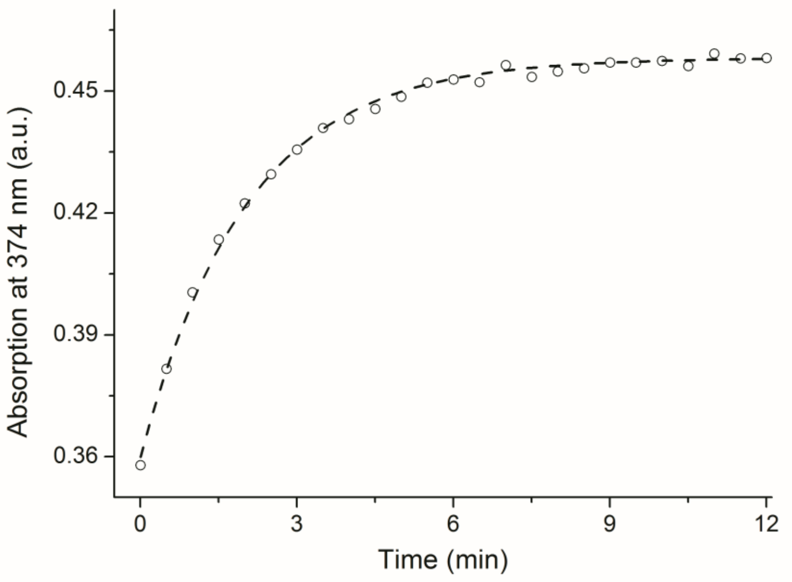

Figure S9. Thermal *Z*-**3** → *E*-**3** (30 µM) kinetics at 37 °C in mQ-water. After 3 min 365 nm light exposure (PSS, t = 0), the sample was placed in the dark. Absorbance at λ = 374 nm (hollow spheres) was monitored over time and fitted to a first order exponential function (dashed line), yielding a time constant for thermal *Z*-**3** → *E*-**3** conversion of τ = 2.0 min.

3.3 Dose-response data for 1

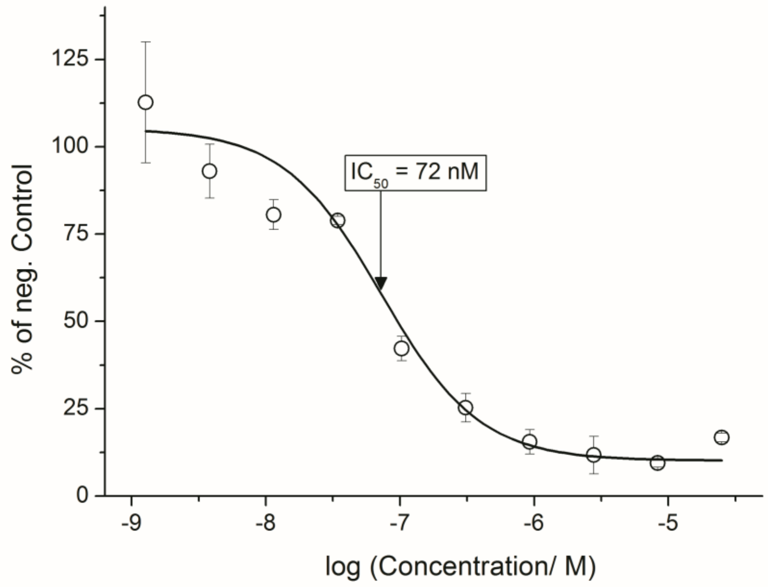

Figure S10. Cell-free RET incubation with **1**. RET-induced ATP turnover was monitored *via* luminescence intensity. The activity readout of **1** (hollow circles) was referenced to a negative control (without inhibitor). Fitting to the Hill-equation renders an IC_{50}-value of 72 nM. Error bars are mean ± standard deviation of duplicate samples.

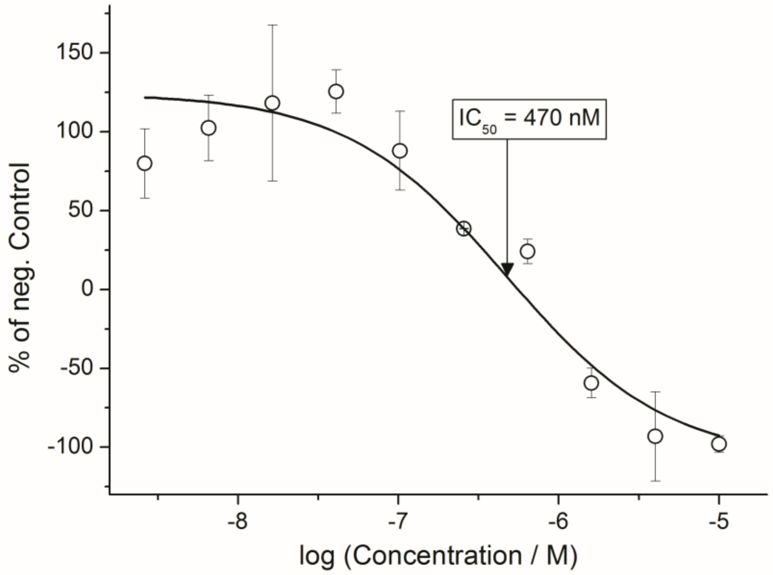

Figure S11. Live-cell RET incubation with **1**. RET-activity was monitored *via* luminescence intensity. The activity readout of **1** (hollow circles) was referenced to a negative control (without inhibitor). Fitting to the Hill-equation renders an IC_{50}-values of 470 nM and. Error bars are mean ± standard deviation of duplicate samples.

3.4 Photo- and thermal kinetics of 4

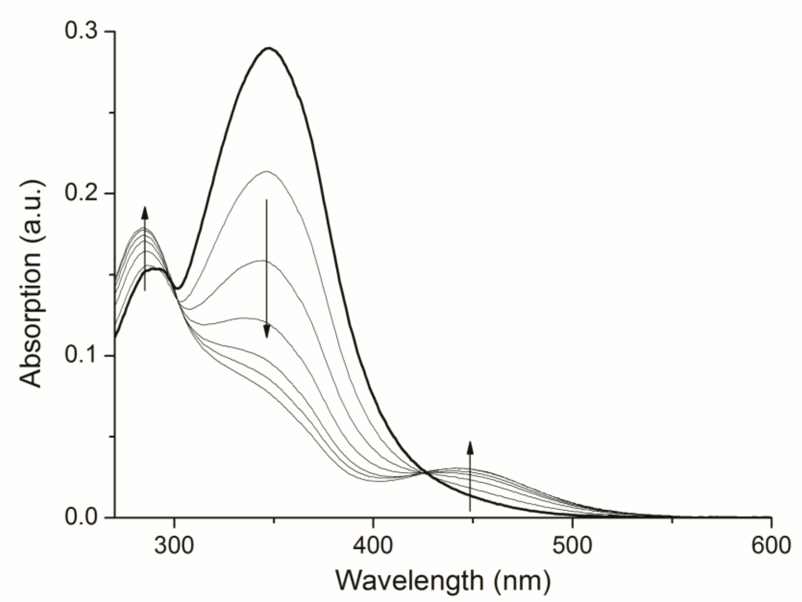

Figure S12. Photoinduced *E*-**4** → *Z*-**4** isomerization of **4** (17 µM) in mQ-water. As-dissolved *E*-**4** (thick line) was successively subjected to 365 nm light for 10 s, 20 s, 30 s, 40 s, 50 s, and 60 s. The photoswitching occurs with isosbestic points at 299 nm and 426 nm.

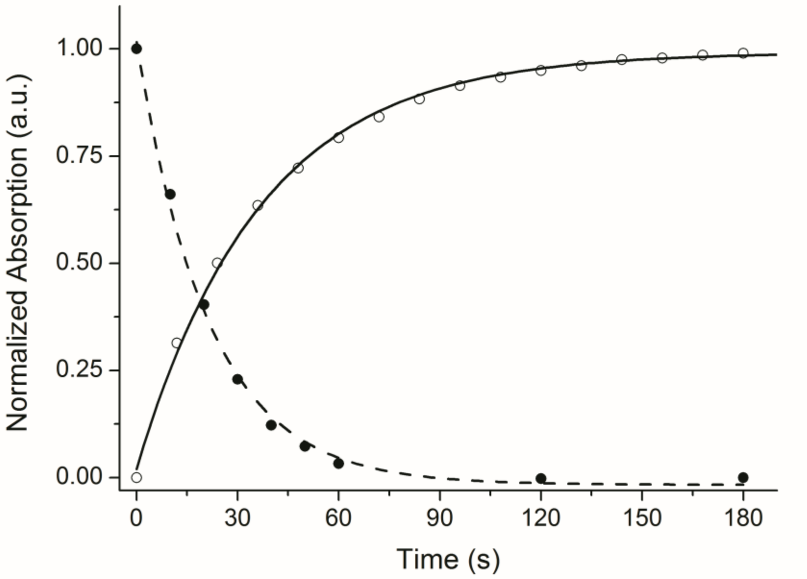

Figure S13. Photoinduced isomerization kinetics of **4** (17 µM) in mQ-water. Absorption at λ = 349 nm for as-dissolved *E*-**4** was monitored (solid circles) during continuous 365 nm irradiation and fitted to a first order exponential function (dashed line, τ = 37 s, 700 µW/cm^2, 100% sample area exposed). Absorption at λ = 349 nm for a *Z*-**4** enriched sample (17 µM) was monitored during continuous 503 nm irradiation (hollow circles) and fitted to a first order exponential function (solid line, τ = 21 s, 14 mW/cm^2, 50% sample area exposed). Both samples were continuously stirred throughout the irradiation.

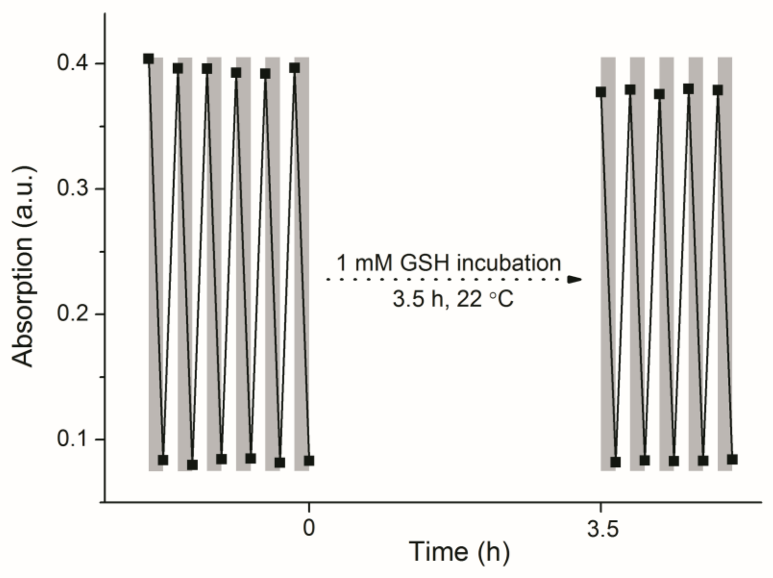

Figure S14. Photoswitching of **4** (22 µM) in the presence of reduced glutathione (GSH) monitored by absorption at λ = 349 nm. First, the as-dissolved *E*-**4** (in 1.0 mM GSH, 10 mM phosphate buffer, pH 7) was subjected to alternating 365 nm (3 min, gray bars) and 503 nm irradiation (3 min, white bars) for a total of 5 cycles. Thereafter, the sample was placed in the dark at 22 °C, 3.5 h. Next, the sample was subjected to an additional 4 cycles of irradiation. The GSH incubation was designed to mimic the live-cell experiment environment. It can clearly be seen that > 95% of the switching capacity is maintained after incubation, *i.e.* no significant GSH-induced degradation occurs.

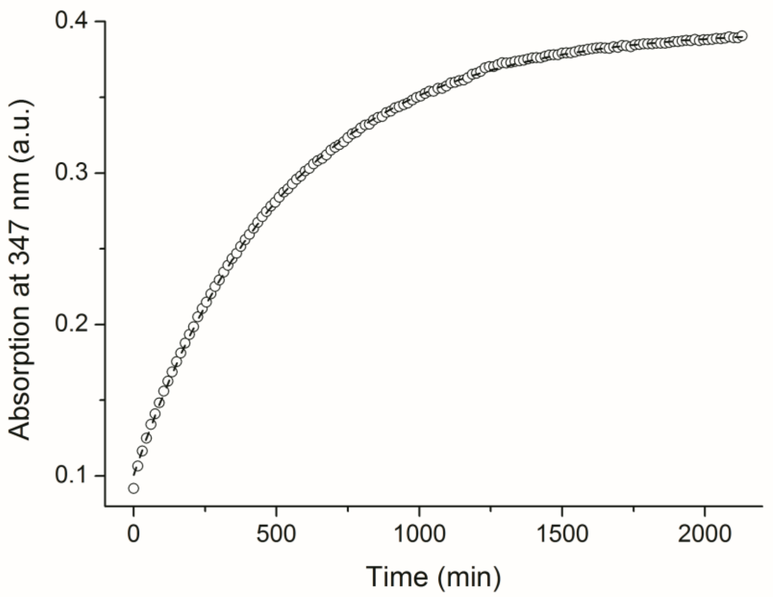

Figure S15. Thermal *Z*-**4** → *E*-**4** (22 µM) kinetics in mQ-water at 37 °C. After 3 min 365 nm light exposure (PSS, t = 0), absorption at λ = 347 nm (hollow circles) was monitored over time and fitted to a first order exponential function (dashed line), yielding a time constant for thermal *Z*-**4** → *E*-**4** conversion of τ = 9.7 h.

3.5 UV-tolerance of RET incubation assays

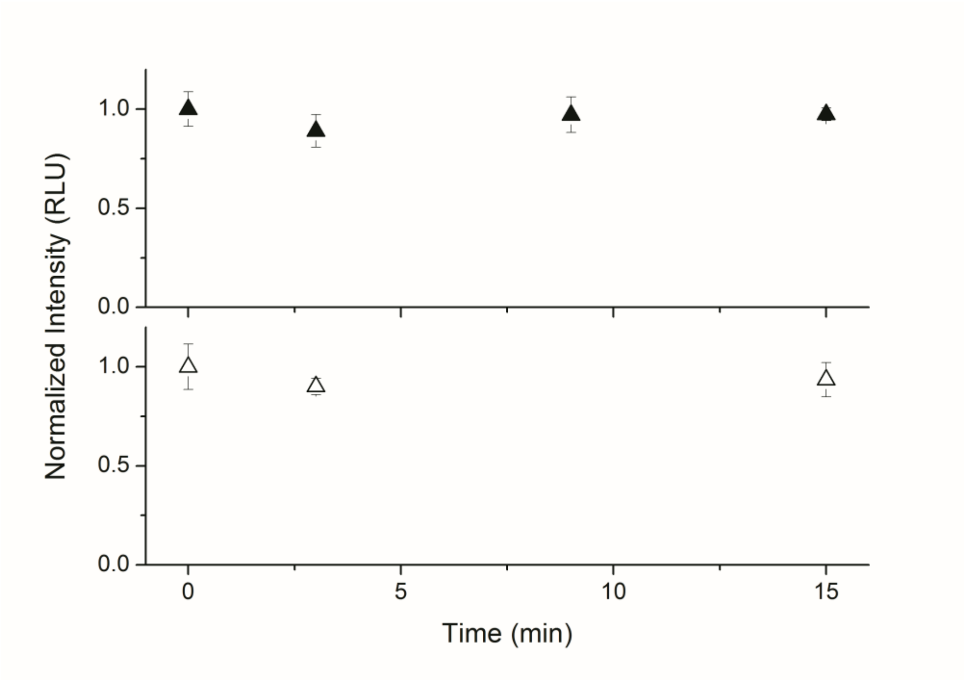

Figure S16. UV-tolerance of RET incubation assays. Top panel: Cell free assay; A reaction mixture comprising RET kinase (0.8 µg/mL) and substrate (40 µg/mL) albeit without inhibitor was subjected to t = 0, 3, 9, or 15 min 365 nm light. Thereafter, ATP was added (50 µM) and the RET kinase activity was assessed (solid triangles). Bottom panel: Live-cell assay; Thawed and acclimatized cells (100 000 cells/mL) without inhibitor was subjected to t = 0, 3, or 15 min 365 nm light. Thereafter, Neurturin was added (at EC_{80}, determined to 15 ng/mL) and the RET kinase activity was assessed (hollow triangles). Error bars are mean ± standard deviation of duplicate samples. It is clear that the applied UV-light has no apparent effect on the enzymatic activity in the cell-free or live-cell assay.

4. References

1. Davoll, J. Pyrrolo[2,3-d]pyrimidines. *J. Chem. Soc. (Resumed).* 131-138 (1960).
2. Dinér, P., Alao, J. P., Söderlund, J., Sunnerhagen, P. & Grøtli, M. Preparation of 3-Substituted-1-Isopropyl-1H-pyrazolo[3,4-d]pyrimidin-4-amines as RET Kinase Inhibitors. *J. Med. Chem.* **55**, 4872-4876 (2012).
